# Degrees of Freedom in Planning, Running, Analyzing, and Reporting Psychological Studies: A Checklist to Avoid *p*-Hacking

**DOI:** 10.3389/fpsyg.2016.01832

**Published:** 2016-11-25

**Authors:** Jelte M. Wicherts, Coosje L. S. Veldkamp, Hilde E. M. Augusteijn, Marjan Bakker, Robbie C. M. van Aert, Marcel A. L. M. van Assen

**Affiliations:** Methodology and Statistics, Tilburg UniversityTilburg, Netherlands

**Keywords:** questionable research practices, experimental design, significance testing, *p*-hacking, bias, significance chasing, research methods education

## Abstract

The designing, collecting, analyzing, and reporting of psychological studies entail many choices that are often arbitrary. The opportunistic use of these so-called researcher degrees of freedom aimed at obtaining statistically significant results is problematic because it enhances the chances of false positive results and may inflate effect size estimates. In this review article, we present an extensive list of 34 degrees of freedom that researchers have in formulating hypotheses, and in designing, running, analyzing, and reporting of psychological research. The list can be used in research methods education, and as a checklist to assess the quality of preregistrations and to determine the potential for bias due to (arbitrary) choices in unregistered studies.

From the inception of the first study idea to the final publication, psychological studies involve numerous choices that are often arbitrary from a substantive or methodological point of view. These choices could affect the outcome of significance tests applied to the data, and hence the conclusions drawn from the research. These choices are also called researcher degrees freedom (Simmons et al., 2011) in formulating hypotheses, and designing, running, analyzing, and reporting of psychological studies, and they have received considerable recent interest for two main reasons. First, researchers’ opportunistic use of them greatly increases the chances of finding a false positive result (Ioannidis, 2005; Simmons et al., 2011; DeCoster et al., 2015), or a Type I error in the language of Neyman–Pearson’s variant of null hypothesis testing (NHST). Second, their strategic use in research may inflate effect sizes (Ioannidis, 2008; Bakker et al., 2012; Simonsohn et al., 2014; van Aert et al., 2016). Hence, researcher degrees of freedom play a central role in the creation of (published) research findings that are both hard to reproduce in a reanalysis of the same data and difficult to replicate in independent samples (Asendorpf et al., 2013).

Among many potential solutions to counter inflated effects and elevated chances of finding false positive results caused by researcher degrees of freedom, one solution has received most attention: preregistration (de Groot, 1956/2014; Wagenmakers et al., 2012; Chambers, 2013). Preregistration requires the researcher to stipulate in advance the research hypothesis, data collection plan, specific analyses, and what will be reported in the paper. Although “planned research” more accurately describes this preregistered research, we will employ the commonly used term “confirmatory research” to describe it. An increasing number of journals now support preregistration for confirmatory research (e.g., Eich, 2014). In addition, over two dozen journals now use a format of registered reports (Chambers, 2013) in which the registrations themselves are subject to peer review and revisions before the data collection starts, and the report is accepted for publication regardless of the direction, strength, or statistical significance of the final results. For instance, this format is now used in the journals *Cortex*, *Comprehensive Results in Social Psychology*, and *Perspectives on Psychological Science* (for Registered Replication Reports).

To disallow researchers to still use researcher degrees of freedom, it is crucial that preregistrations provide a *specific*, *precise*, and *exhaustive* plan of the study. That is, the ideal preregistration should provide a detailed description of all steps that will be taken from hypothesis to the final report (it should be specific). Moreover, each described step should allow only one interpretation or implementation (it should be precise). Finally, a preregistration should exclude the possibility that other steps may also be taken (it should be exhaustive). Hence, a preregistration specifies the project in such a way that all potential contingencies in formulating hypotheses, and designing, running, analyzing, and reporting are covered. For instance, the syntax for the statistical analyses should preferably be created in advance to be run (once) on the collected data to yield the final statistical results. Our own experiences with preregistration taught us that this specification is no easy task and that maneuverability remains if preregistrations are not sufficiently specific, precise, or exhaustive. For instance, just indicating one’s use of a certain scale as the main outcome measure in an experiment typically does not preclude the researcher to attempt many different ways in how to score the items of the scale in his or her pursuit for statistical significance. A preregistration should also be exhaustive because the stipulation that one will test Hypothesis A in a certain way does not preclude the possibility that one can *also* test Hypothesis B in the study. Therefore, for confirmatory aspects of the study, the word “only” is key (e.g., “we will test only Hypothesis A in the following unique manner”).

The goal of this paper is to present a list of researcher degrees of freedom that can be used in research methods education, as a checklist to assess the quality of preregistrations, and to determine the potential for bias due to (arbitrary) choices in unregistered studies. By pointing out many different researcher degrees of freedom, we hope to raise awareness of the risk of bias implicit in a lot of research designs in psychology and beyond. The list enables a charting of what Gelman and Loken (2014) dubbed the garden of forking paths in the analysis of data; i.e., the many different analytic decisions that could be or could have been made with a given data set. In what follows, we use the singular term researcher DF (degree of freedom) to mean a particular choice during the study, and the plural term researcher DFs when referring to multiple researcher degrees of freedom (or different types of choices).

Because NHST is by far the most used statistical framework used in psychology and related fields, we created the list of researcher DFs with NHST in mind. Other possible statistical frameworks are based on confidence intervals (Cumming, 2012), precision of effect size estimation (Maxwell et al., 2015), or Bayesian statistics (e.g., Kruschke, 2015). We note that most researcher DFs are relevant for all statistical frameworks. However, some researcher DFs need to be replaced or omitted (e.g., power analysis [D6], which is defined in the NHST framework) or added (e.g., selection of the prior, which is only used in Bayesian statistics) in approaches other than NHST. At this point, we therefore recommend using our list primarily for research using NHST.

We created the list in a qualitative manner; we as a group of methodologists studying researcher DFs, publication bias, meta-analysis, misreporting of results, and reporting biases, came up with a large list of researcher DFs, discussed these, and created a manageable list in several rounds of revision. We are aware that our list may not be exhaustive, but believe the list is a good starting point for a checklist that serves the purpose to assess the degree to which preregistrations truly protect against the biasing effects of researcher DFs. Most of the researcher DFs have been described in previous publications (Kriegeskorte et al., 2009; Nieuwenhuis et al., 2011; Simmons et al., 2011; Wagenmakers et al., 2011; Bakker et al., 2012; John et al., 2012; Chambers, 2013; Francis, 2013; Bakker and Wicherts, 2014; Simonsohn et al., 2014; Steegen et al., 2016; van Aert et al., 2016). The researcher DFs on our list are invariably inspired by actual research we have encountered as researchers, replicators, re-analyzers, and critical readers of published and unpublished works. Although we have actual examples of the use of each of the researcher DFs we discuss below, we choose not to identify the researchers, projects, or papers involved; the issues are general and it would not help to focus on individual cases.

We discuss the different researcher DFs categorized under headers that refer to different phases in a study from its early theoretical underpinnings to its final publication (hypothesizing, designing, data collection, analyzing, and reporting), and indicate links between different researcher DFs at the different phases. Each researcher DF will be coded according to its phase. All researcher DFs are listed in **Table 1**. We focus on experiments as the most basic design used to study the (causal) effect(s) of independent variable(s) on the dependent variable(s). This design is the most widely used in psychology and entails a good archetype to discuss the many researcher DFs in a multitude of research designs, including quasi-experimental studies and correlational studies aimed to explain dependent variables on the basis of one or more predictor variable(s).

**Table 1:** Checklist for different types of researcher degrees of freedom in the planning, executing, analyzing, and reporting of psychological studies.

Code	Related	Type of degrees of freedom
Hypothesizing
T1	R6	Conducting explorative research without any hypothesis
T2		Studying a vague hypothesis that fails to specify the direction of the effect
Design
D1	A8	Creating multiple manipulated independent variables and conditions
D2	A10	Measuring additional variables that can later be selected as covariates, independent variables, mediators, or moderators
D3	A5	Measuring the same dependent variable in several alternative ways
D4	A7	Measuring additional constructs that could potentially act as primary outcomes
D5	A12	Measuring additional variables that enable later exclusion of participants from the analyses (e.g., awareness or manipulation checks)
D6		Failing to conduct a well-founded power analysis
D7	C4	Failing to specify the sampling plan and allowing for running (multiple) small studies
Collection
C1		Failing to randomly assign participants to conditions
C2		Insufficient blinding of participants and/or experimenters
C3		Correcting, coding, or discarding data during data collection in a non-blinded manner
C4	D7	Determining the data collection stopping rule on the basis of desired results or intermediate significance testing
Analyses
A1		Choosing between different options of dealing with incomplete or missing data on *ad hoc* grounds
A2		Specifying pre-processing of data (e.g., cleaning, normalization, smoothing, motion correction) in an *ad hoc* manner
A3		Deciding how to deal with violations of statistical assumptions in an *ad hoc* manner
A4		Deciding on how to deal with outliers in an *ad hoc* manner
A5	D3	Selecting the dependent variable out of several alternative measures of the same construct
A6		Trying out different ways to score the chosen primary dependent variable
A7	D4	Selecting another construct as the primary outcome
A8	D1	Selecting independent variables out of a set of manipulated independent variables
A9	D1	Operationalizing manipulated independent variables in different ways (e.g., by discarding or combining levels of factors)
A10	D2	Choosing to include different measured variables as covariates, independent variables, mediators, or moderators
A11		Operationalizing non-manipulated independent variables in different ways
A12	D5	Using alternative inclusion and exclusion criteria got selecting participants in analyses
A13		Choosing between different statistical models
A14		Choosing the estimation method, software package, and computation of SEs
A15		Choosing inference criteria (e.g., Bayes factors, alpha level, sidedness of the test, corrections for multiple testing)
Reporting
R1		Failing to assure reproducibility (verifying the data collection and data analysis)
R2		Failing to enable replication (re-running of the study)
R3		Failing to mention, misrepresenting, or misidentifying the study preregistration
R4		Failing to report so-called “failed studies” that were originally deemed relevant to the research question
R5		Misreporting results and *p*-values
R6	T1	Presenting exploratory analyses as confirmatory (HARKing)

## Hypothesizing Phase

The degree to which the researcher can find relevant statistically significant results in the data is already partly determined by the specificity of theoretical predictions that the study aims to address. A confirmatory study requires a clearly stated hypothesis to be tested, while more exploratory studies aimed at finding interesting (typically statistically significant) patterns in the data often lack *a priori* theorizing on (causal) relations. Such exploratory studies are virtually guaranteed to yield support for something interesting (Wagenmakers et al., 2011). Since most results in psychology are presented in the realm of the hypothetico-deductive model (Hubbard, 2015), it is tempting to present exploratory findings incorrectly as having been hypothesized in advance. This practice, to which we return when discussing the reporting phase, is also called Hypothesizing After Results are Known or HARKing (Kerr, 1998). The relevant researcher DF during the theorizing phase, namely *T1: Conducting explorative research* pervades many of the researcher DFs that we describe below in the later phases of the study. HARKing yields statistical evidence for found patterns that is often much weaker than it appears to be. The reason is that the evidence should be seen in the context of the size and breadth of the explorations, allowing for appropriate corrections for multiple testing. Unfortunately, data explorations are often presented without such necessary corrections. T1 could be dealt with by specifying the independent variable and the dependent variable of interest before running the study, preferably in a preregistration of the study (Wagenmakers et al., 2012). Note that even a preregistered (confirmatory) study can include some exploratory analyses, which is unproblematic as long as these explorations are clearly distinguished from the confirmatory analyses.

However, even if there is a vague prior notion about the relation between the independent and dependent variables, hypotheses that fail to indicate the direction of an effect (or relation) enable later flexibility in the analysis and interpretation of results (Schaller, 2016). If the hypothesis is merely “X is related to Y” or “X affects Y,” the researcher can later analyze the data in two alternative ways; one way to obtain a positive effect and another way to obtain a negative effect of X on Y, which entails a strategy that is a special case of HARKing. The researcher DF is *T2: Studying a vague hypothesis that fails to specify the direction of the effect.* Note that specifying the direction of the hypothesized effect is relevant for the decision to use a one- or two-tailed test. One-tailed tests can only be used to reject the null hypothesis when the *a priori* hypothesis was directional and the result was in the predicted direction. Testing hypotheses requires specificity and precision regardless of whether one uses one- or two-tailed tests. Consequently, a preregistered hypothesis needs to specify the direction of the effect or relation. Because of the need for proper power analyses (discussed below under D6), it is also important to have a prior sense of the size of the effect or strength of the relation.

## Design Phase

Although most researcher DFs discussed in the literature pertain to the analysis of the data, both the theoretical predictions and the design of an experiment (or other types of studies) already allow the researcher to create options for flexible analyses in later phases of the study. A psychological experiment can be set up to have a certain degree of redundancy in the design that creates maneuverability in the collection of data, analysis of data, and reporting of results. This redundancy applies to both independent variables and dependent variables.

### Independent Variable(s)

We distinguish here between manipulated and non-manipulated independent variables. Manipulated independent variables are those manipulated in the design of the experiment, and typically involve randomization. In contrast, non-manipulated independent variables are based on measures of behavior or individual differences that (could) pertain to the research question. These independent variables are the main focus in correlational studies, but are also widely used in studying (moderation of) experimental effects. Moreover, additional measures taken after the manipulation or in correlational studies could later be used as mediators in explaining variance in the dependent variable, but an underspecified preregistration enables researchers to use these variables as primary dependent variable as well (see our Discussion of D4).

Experiments can involve multiple manipulated independent variables (i.e., experimental factors), that are often crossed and that researchers can select or discard in later analyses based on particular (preferred) outcomes. Dropping of experimental conditions has been found to be quite common in a survey among psychological researchers (John et al., 2012) and in a study that considered psychological studies from a register (Franco et al., 2016). Specifically, a researcher can discard a factor in a multifactorial experiment by pooling the data over the levels of that factor, or the researcher can select certain levels of a discarded factor. For instance, in a two-by-two factorial design studying the effects of both ostracism (including vs. excluding someone socially) and group composition (being in- or excluded by either a social in-group or a social out-group) on participants’ mood, the researcher could ignore group composition either by pooling in- and outgroup levels, or by selecting one of the levels of group composition (say, the in-group) in the later analyses. Moreover, a given experimental factor involving more than two levels can later be analyzed in different ways. For instance, if an experimental factor has three conditions (say, 0, 1, and 2), the researcher can focus on all three levels, but also select two out of the three in the later analyses. Or the researcher can combine conditions 0 and 1 to compare it with condition 2, etc. In this way, this simple three level factor already yields seven different operationalizations for the analysis, from which the researcher can later choose one(s) that yielded the “best” result. So the design of manipulated independent variables offers the following researcher DF*: D1 creating multiple manipulated independent variables and conditions.* Like all researcher DFs, this researcher DF becomes more relevant as the number of scoring options increases, like with complex mixed designs involving multiple between-subject and within-subject factors featuring multiple levels. Consequently, preregistrations of such studies should specifically and precisely delineate how independent variables are later used in testing the focal hypotheses.

Non-manipulated independent variables based on the observed characteristics of participants, are also measured in most research designs. These non-manipulated independent variables such as personality characteristics, IQ, age, gender, ethnicity, political preference, etc. offer great flexibility in the later analyses of the data; one can use them as main predictor, but also as moderators to study potential interactions with manipulated factors, or as control variables (covariates) as in ANCOVA. For instance, measured age can assume any of these roles in later analyses: e.g., for studying age differences, for testing whether age moderates the effects of any of the other independent variable(s), or as a control variable to explain some of the within-condition variation in the dependent variable. Moreover, measures taken after the manipulations can be used in later mediation analyses to explain variance in the dependent variable. This entails *D2: Measuring additional variables that can be selected later as covariates, independent variables, mediators, or moderators*. Obviously, adding more measures offers multiple ways to find interesting patterns in later stages of the study. Just as manipulated independent variables can often be operationalized in different ways, many non-manipulated independent variables, once selected, offer flexibility in how they will be used in the analyses. Participants can be assigned to different levels of those independent variables on the basis of flexible thresholds or category assignments (Steegen et al., 2016). For instance, age can be used to create two age groups (young and old), or three age groups (young, middle-aged, and old) on the basis of many different age-based category assignments. However, age can also be used as a continuous factor, covariate or moderator in later analyses. Similar flexibility applies to designs that involve demographic variables (e.g., income, SES, educational level, ethnicity, mother tongue, relationship status) or psychological individual differences (e.g., IQ, extraversion, diagnostic criteria, etc.) and is discussed below in the context of the analyses.

In sum, a research design that is littered with research DFs related to independent variables is complex and offers room for selecting and operationalizing these variables in multiple ways. An ideal preregistration, then, specifically and precisely specifies which manipulated independent variables and non-manipulated independent variables will be used in the analyses and also indicates how both types of variables are to be operationalized, and that no other variables are to be used in the confirmatory analyses. We again emphasize that these specifications are only necessary for the confirmatory analyses; a potential exploratory analyses section of a paper is not at all problematic, as long as the preregistration and the paper clearly distinguish between these very different types of analyses.

### Dependent Variable(s)

The measurement of human behavior is often complex and is seldom done in a single predefined manner. A design prone to bias due to researcher DFs offers multiple dependent measures of the same construct. This enables the researcher to choose among different outcome measures the one(s) that offer(s) statistical significance. The relevant researcher DF in the design phase is *D3: Measuring the same dependent variable in several alternative ways.* For instance, anxiety can be measured with various self-report scales, or with physiological measures (e.g., galvanic skin response, heart rate variability).

Another particularly flexible design allows the researcher to choose among several dependent variables that concern *different* constructs in cases where the originally targeted primary outcome failed to show statistically significant effects. Among the research practices studied by John et al. (2012) this practice of not reporting all dependent measures showed quite high prevalence estimates. Additionally, direct evidence indicates that psychological researchers often choose among different outcome measures (LeBel et al., 2013; Franco et al., 2016). In the medical literature on randomized clinical trials, this researcher DF is often called outcome switching and the bias it introduces is called outcome reporting bias (Chan et al., 2004; Kirkham et al., 2010; Weston et al., 2016). For instance, outcomes that were initially designated as secondary outcome variables appeared as primary outcome variables in the published article. Or a variable that was originally viewed as a potential mediator of an effect might replace the original main outcome variable if the latter failed to show an effect. Here we denote this researcher DF by *D4: Measuring additional constructs that could potentially act as primary outcomes.*

Thus in the design of studies, the researcher can already create many researcher DFs that allow for opportunistic use in later phases of the research process, relating to using multiple measures of the same construct (D3), and creating opportunities to find additional effects by adding measures of additional constructs besides the one(s) that were the original focus of interest (D4). D4 allows HARKing (Kerr, 1998), whereas D3 is aimed at the same targeted construct and related to how the primary outcome will be used in later analyses. It is clear that the ideal preregistration should specify which dependent variable(s) will be used in testing particular hypotheses. However, as we discuss below, even specifying the measure (say, the Rosenberg Self-Esteem Scale) that is to be used as primary outcome is *not* specific and precise enough to avoid *p*-hacking during analyses, because often the scores on such measures can be computed in different *ad hoc* ways.

### Excluding Participants

Adding numerous measures besides the main independent and dependent variables to the design offers yet another researcher DF: background variables (e.g., age, gender, ethnicity) or other individual differences can be used to later discard participants in an *ad hoc* manner from the analysis. For instance, a researcher might decide to exclude older-aged participants for some reason that might actually not be independent of the effect of the exclusion on the final analysis. Such exclusion of cases on the basis of measured variables often comes across as *ad hoc* because if that decision rule had been *a priori*, these older-aged participants should not have completed the study in the first place.

Other types of measures that can be used to discard participants include awareness checks, as often used in priming research (e.g., the funnel debriefing; Bargh and Chartrand, 2000), checks for alertness in responding like the blue dot task (Oppenheimer et al., 2009), or even the simple question like “did you participate seriously in this study?”. Decision rules in how to deal with these questions need to be pre-specified to avoid them becoming a researcher DF. Similarly, manipulation checks (i.e., measures of the independent variable) can also be implemented in the design, offering a way not only to assess the strength of the manipulation, but also to discard particular participants from the analyses for not showing any desired response to the manipulation. These decisions in the data selection offer great flexibility in choosing whom to include in the analysis. *D5: Measuring additional variables that enable later exclusion of participants from the analyses (e.g., awareness and manipulation checks).* Therefore, an ideal preregistration specifically and precisely describes which types of participants will be excluded from the analyses, and also explicates that the stated rules of exclusion will be the only ones that will be used to discard participants (it should be exhaustive). The reason is that only stating a particular exclusion rule in the preregistration does not preclude the possibility to also exclude participants on other *ad hoc* grounds.

### Power and Sampling Plan

Despite the core importance of statistical power in NHST, most studies using NHST fail to report a formal power analysis (Sedlmeier and Gigerenzer, 1989; Cohen, 1990; Bakker et al., 2012). This is problematic because researchers’ intuitions about power are typically overly optimistic (Bakker et al., 2016) and studies in psychology are often underpowered. More importantly, underpowered studies are themselves more susceptible to bias (Bakker et al., 2012), particularly in combination with the use of many of the other researcher DFs that we describe here. The reason is that the sampling variability is larger and many decisions made in analyzing the data will have proportionately larger effects when sample sizes are smaller. In other words, using researcher DFs to obtain statistically significant results is typically more effective with smaller samples. Low power can create bias and hence D6: *Failing to conduct a well-founded power analysis* is a researcher DF in designing studies.

A rigorous preregistration not only provides a good rationale for the sample size in the form of a power analysis, but also should describe the complete sampling plan, i.e., the targeted sample size, when the data collection starts and ends, and how the participants are to be sampled. The sampling plan should specify the population from which is sampled, the procedure of sampling, and the end point of data collection. The sampling plan should also specify when additional participants are to be sampled in cases where the targeted sample size is not met (e.g., due to drop-out or data exclusions). The sampling plan should be specific and precise to disallow researchers to conduct intermediate tests during data collection. If not, a researcher can decide to collect more data after witnessing a non-significant result or to cease data collection earlier than planned if the result is already significant (see also C4), both of which affect the Type I error rate. The sampling plan should also preclude the researcher to conduct a particular study multiple times, and only present the “best” study (i.e., the one with the most desirable results). The use of multiple small studies instead of a larger one is an effective (yet problematic) strategy to find at least one statistically significant result (Bakker et al., 2012) and small underpowered studies can also be pooled by means of a meta-analysis in an *ad hoc* manner to obtain a statistically significant result (Ueno et al., 2016). Hence, we call the following researcher DF, *D7: Failing to specify the sampling plan and allowing for running (multiple) small studies.*

## Data Collection Phase

During the collection of experimental data, it is possible to act in certain ways that enhance the probability of finding a statistically significant result. Most of the issues are methodological, although some are statistical and bear on issues of multiple testing and sequential analyses. In our discussion, we assume that the design itself is internally valid and that the measures are construct valid, in the sense that the experiment does not involve any confounds or artifacts and uses appropriate measures. This, of course, does not always mean that the actual study does not suffer from threats to internal validity or construct validity.

### Non-random Assignment

Although methodological textbooks are clear on the benefits of random assignment, the randomization techniques used to assign participants to conditions are often not specified in research articles. Using non-random assignment could greatly affect differences between conditions in personal characteristics or other factors that could affect the outcome. For instance, an experimenter might (purposively or not) only run treatment participants in the evening, thereby creating a potential confound, or the assignment could be based on observable personal characteristics that might bear on the outcome measure (e.g., a particularly slow moving participant is assigned to the condition that aligns with slowness). In other words, the randomization technique should be specifically and precisely stipulated in advance and followed throughout the experiment, thereby avoiding *C1: The failure to randomly assign participants to conditions.*

### Incomplete Blinding

It is widely recommended to employ double-blinding techniques to avoid demand characteristics and placebo effects on part of participants as well as experimenter expectancy effects during data collection (Rosenthal, 1966). Participants are blinded if the design prevents them from knowing to which condition they have been assigned or from knowing the research hypotheses. Experimenters are blinded if they do not know to which condition a participant is allocated at any time. There are several ways in which both types of blinding can be unsuccessful, potentially leading experimenters to treat participants (unwillingly) differently across conditions, or participants to act in ways that yield invalid support for the research hypothesis. Hence, *C2: Insufficient blinding of experimenters and/or participants* could potentially introduce bias. For instance, experimenters could use non-naïve participants (e.g., a fellow student) or (in)avertedly convey information about what is expected from participants in a given condition. The preregistration study should specifically and precisely describe the procedure of how participants and experimenter(s) are blinded, if applicable.

### Discarding, Correcting and Coding Data

If experimenters are involved in coding or other ways of data handling, incomplete blinding concerning condition assignment or hypotheses could introduce bias. Working with participants is a social process in which experimenters has information about participants or their behavior that might enable them to predict scores on the dependent variable for individual participants. For instance, an experimenter may witness a slowly working student in a condition that is expected to yield quick responses and might decide to discard that participant for not participating seriously even though there is no clear experimental protocol that dictates such a decision. This creates biases during the data collection, and such biases are particularly problematic in experiments involving coding of behavior in a non-blinded manner. Similarly, missing values or incorrectly filled out answers on a questionnaire or test could also be corrected or filled out during data collection by someone who is not blind to condition (or the hypotheses) and hence might make biased decisions. For instance, the experimenter could decide to correct or fill in the answer of a participant who indicated the incorrect gender or no gender on a questionnaire. Although making such corrections or imputations deliberately might go beyond questionable and so might entail falsification (a violation of research integrity), doing this without awareness in a poorly structured research setting might nonetheless cause considerable bias. A specific, precise, and exhaustive research protocol can help avoid this researcher DF. *C3: Correcting, coding, or discarding data during data collection in a non-blinded manner.*

### Intermediate Significance Testing

The decision whether or not to continue with data collection could be dependent on intermediate analyses of the data. This is reflected by the common practice to continue data collection after witnessing a statistically non-significant result or by quitting data collection earlier than planned after witnessing a statistically significant result (John et al., 2012). It is well known that this type of sequential testing is problematic without any formal correction for multiple testing (Wagenmakers, 2007) and increase Type 1 error rates. *C4: Determining the data collection stopping rule on the basis of desired results or intermediate significance testing.* A specific and precise *a priori* sampling plan could ameliorate this, and so this researcher DF is related to D7 described above.

## Analysis Phase

In the analysis phase, the researcher directly witnesses the effects of choices on the statistical outcome. It is surprising that blinding to conditions and hypotheses of experimenters, coders, and observers is considered to be crucial during data collection, while in practice, the analyses are typically conducted by a person who is not only aware of the hypotheses, but also benefits directly from corroborating them (commonly by means of a significance test). Together with the many researcher DFs during the analyses, these factors do not entail the most optimal mix for objective and unbiased results.

### Data Cleaning and Processing

Before running the focal analyses, experimental data often need to be cleaned and prepared for analysis. Data cleaning involves many choices related to missingness, outliers, or violations of the distributional assumptions. Because of potential drop-out, data collection problems, or a lack of full responses for other reasons (e.g., participants’ inattention or refusal to answer some questions), some data might be missing entirely for participants or for some or many of the variables of interest. Missing data can be dealt with by listwise deletion, pairwise deletion, multiple imputation, full information methods, and other methods (Schafer and Graham, 2002). This choice creates a researcher DF, namely *A1: Choosing between different options of dealing with incomplete or missing data on ad hoc grounds.*

Neuroimaging techniques (e.g., signals from fMRI, EEG, MEG) and other data-intense measurement procedures require extensive pre-processing steps that entail considerable maneuverability in the analysis of the data (Kriegeskorte et al., 2009, 2010; Poldrack et al., 2016). For instance, with neuroimaging data, decisions related to regions of interest, dealing with head motions, corrections for slice timing, spatial smoothing, and spatial normalization can create a large number of different ways to analyze the data (Poldrack et al., 2016). The processing of such data can be done based on whether they provide preferred results, which offers *A2: Specifying pre-processing of data (e.g., cleaning, normalization, smoothing, motion correction) in an ad hoc manner.*

Tests have assumptions related to how the data are distributed. The typical assumption in the *F*-family of parametric tests is that the data are independently normally distributed and that variances of different groups are homogenous. There are various ways to deal with violated assumptions of such statistical tests: one could use non-parametric analyses, transform the data in various ways to approach normality or simply ignore the violations. Moreover, violations of variance homogeneity in ANOVAs or *t*-tests, non-normality, non-linearity in linear models, or heteroscedasticity in regression could be dealt with in several alternative ways (Wilcox, 2012). When done in a data-driven manner, this creates: *A3: Deciding on how to deal with violations of statistical assumptions in an ad hoc manner.*

Dealing with outliers is a particularly vexing issue that warrants specifically, precisely, and exhaustively described protocols in a preregistration. Outliers can be operationalized and detected in various ways (Barnett and Lewis, 1994; Wilcox, 2012; Bakker and Wicherts, 2014) and they can be deleted or kept on the basis of many alternative criteria that could be chosen based on whether they lead to significance. Alternatively, the researcher can choose to conduct analyses that are less sensitive to outliers, like non-parametric or robust analyses. This creates *A4: Deciding on how to deal with outliers in an ad hoc manner.*

### Dependent Variable(s)

Statistical analyses of experimental data boil down to predicting scores on the outcome measure chosen in the analysis on the basis of predictors (typically factors, but also covariates, mediators, and/or interaction terms). While running the analysis, the researcher can choose between different measures or operationalizations of the same construct implemented in the design of the study in an effort to find the measure that shows the preferred or best results. This practice, which is paired with the use of various measures in the design (D3), concerns the following researcher DF: *A5 selecting the dependent variable out of several alternative measures of the same construct.*

The dependent variable, once selected, can often be scored or operationalized in various ways. There also exist degrees of freedom even if there is only one overall measure or scale. Discarding, weighting, selecting, or redefining scoring rules of individual items can offer flexibility in analyses even if the items are based on a commonly used scale. For example, items of a scale that are originally measured on a five-point Likert scale can be dichotomized, or some items might be discarded from the scale score for showing low or negative item-rest correlations. Moreover, the scale score can be based on an unweighted sum of item scores or on weighting of items based on an item response model, or by running a principal components analysis and choosing among alternative ways to estimate the factor scores. So even for existing scales, flexibility exists in operationalizing the scores in the analyses. The use of response time data involving responses to many stimuli also involves many choices in dealing with slow response times, and how to summarize the major outcome variable. The researcher DF is *A6: Trying out different ways to score the chosen primary dependent variable.*

Finally, researchers can choose to measure additional constructs next to the one(s) originally targeted as the main dependent variable (or primary outcome) in the design (see D4). During the analyses this creates *A7: Selecting another construct as the primary outcome*.

### Independent Variables

If we consider the ANOVA as a regression model, the use of independent variables means selecting among numerous predictors and/or interaction terms to predict the outcome, and hence different regression models. Without specific preregistration, a researcher often has numerous options to choose between different regression models. The researcher can also typically operationalize the non-manipulated and manipulated in various ways, particularly in flexible designs. During the analysis, the researcher can employ *A8: Select independent variables out of a set of manipulated independent variables (paired with D1).* Similarly, even for a given manipulated variable, the researcher can often choose to discard or combine different levels of factors, which creates *A9: Operationalizing the manipulated independent variables in different ways (e.g., by discarding or combining levels of factors; paired with D1).*

Furthermore, during the analyses, the researcher can make opportunistic use of a host of additional non-manipulated measures (D2), as well as possible mediator variables measured during the study, thereby creating *A10: Choosing to include different measured variables as covariates, independent variables, mediators, or moderators* in the analysis. The number of different analytic options considered for finding some statistically significant result (mediation, moderation, main effect) on the basis of measured variables can be quite large. For instance, adding big five personality measures to a simple one-way experimental design enables the researcher to seek for the moderation of effects by all of these personality traits. However, these big five traits can also be used as covariates, or simply as independent variables to help explain variance in the outcome measure(s). More degrees of freedom are added if the researcher does not specifically and precisely describe in advance how these measured variables are to be used and scored in the analysis (Steegen et al., 2016). For example, a measure of extraversion could be used as a linear predictor based on unweighted sum of individual item scores or some estimate of the factor score reflecting the underlying construct. However, the researcher can also compare participants with some (arbitrarily chosen) high or low score on the scale used to measure extraversion (and even there, the researcher could discard some items because they showed low item-rest correlations). This creates *A11: Operationalizing non-manipulated independent variables in different ways.*

An exceptionally flexible analysis involves many different regression models based on a host of different combinations of predictors (main effects, interactions, control variables or covariates), and alternative ways to operationalize these predictors, leading to a very large number of regressions (Sala-I-Martin, 1997) in some designs. For instance, a researcher might add age as a moderator during the analysis and check whether different ways to categorize age groups yields some interesting results. Running so many regressions creates a massive multiple testing problems that can be solved in statistical ways or with a sufficiently detailed preregistration.

### Selection Criteria

One can also change the analysis by altering the sample size on the basis of different criteria to (de)select participants. This yields *A12: Use of alternative inclusion and exclusion criteria in selecting participants for use in the analysis*. This researcher DF is paired with D5, i.e., the design choice to include many additional variables related to manipulation checks or awareness checks or any other personal characteristics that can be used as selection criteria. There are many bases to select or deselect participants for the analysis, including performance (e.g., many alternative levels of the percentage of items answered correctly on some task that measures the manipulation), awareness questions, or any personal characteristics. A specific, precise, and exhaustive plan to not include particular participants in the final data analyses not only avoids this researcher DF, but could also spare resources by simply not collecting any (additional) data for participants who fail to meet the inclusion criteria. For instance, if a linguistics researcher is not interested in participants who are not native speakers of the language of interest, he or she would be better off not running these participants at all, instead of excluding their data only at the analysis phase.

### Statistical Model, Estimation, and Inference

Even for relatively straightforward experiments, many different statistical models can be used to analyze experimental data, including linear regression, ANOVA, MANOVA, or robust or non-parametric analyses. Hence, an obvious researcher DF is *A13: Choice of the statistical model*. However, choosing the statistical model (say, a regression with three predetermined predictors), often does not preclude additional statistical choices. Specifically, statistical models need to be estimated and this can frequently be done in several ways. Even with a given estimation method, the researcher can choose between different corrections to the standard errors (SEs) of parameters. For instance, one could choose for robust SEs instead of the standard SEs. Moreover, different statistical software packages (e.g., SPSS, R, SAS) often implement the same estimation techniques and correction methods in slightly different ways, leading to diverging results. These alternative estimation methods, software packages, and correction methods might lead to different outcomes and hence entail a researcher DF: *A14: The choice for estimation method, software package, and computation of SEs.* To wit, even a standard ANOVA requires a choice between different types of sum of squares, three of which are available in SPSS (this choice is typically not described in articles). This problem is particularly vexing for more advanced analyses, that can be estimated with Maximum Likelihood, Ordinary Least Squares, Weighted Least Squares, Mean and Variance Adjusted Weighted Least Squares, Partial Least Squares, or Restricted ML, with or without robust SEs (to name just a few options).

Finally, without a specific and precise registration, a researcher can choose inference criteria (e.g., Bayes factors, alpha level, sidedness of the test, and corrections for multiple testing) in different ways, and on the basis of analytic outcomes. Thus *A15: Choosing inference criteria (e.g., Bayes factors, alpha level, sidedness of the test, and corrections for multiple testing)* is another researcher DF. For instance, a researcher can choose to use a one-sided test if this is the only way to obtain significance, or employ more lenient corrections for multiple testing if the need arises. Preregistrations should explicate these criteria.

## Reporting Phase

In the reporting of results, the researcher is faced with the freedom to report details of the *a priori* hypotheses, design, data collection, and analysis of the study. Here, the potential exploitations of the many researcher DFs discussed above can or cannot be reported, which renders the reporting of such details crucial (Wigboldus and Dotsch, 2016). For instance, the researcher could report only a subset of many analyses that showed the researcher’s most desirable results. The comprehensive reporting of the study design and results is necessary for both reproducibility (reanalyzing the study using the same data) and replicability (rerunning the study as similar as possible in a new sample) (Asendorpf et al., 2013). It is obvious that the many researcher DFs can be hidden for readers (or critical reviewers) by failing to reporting some independent variables, some dependent variables, missing data, data exclusions, or other relevant choices made during the analyses. Reproducibility requires a verification of the steps taken from the data collection to the final report, including choices made during the collection and analysis of the data, such as pre-processing of the data, the statistical model, the estimation technique, software package, and computational details, data exclusions, dealings with missing or incomplete data, violated distributional assumptions, and outliers. This offers the following researcher DF in the reporting phase: *R1: Failing to assure reproducibility (verifying the data collection and data analysis).* The preferred way to assure reproducibility is to share data and analytic details (computer syntaxes/code) in or alongside the paper (Nosek et al., 2015).

The exploitation of researcher DFs creates bias, which might lower replicability of earlier results in novel samples (Open Science Collaboration, 2015). To allow later (direct) replications of a study, it is crucial that the report (or its supplements) includes sufficient details on the data collection, including procedures and all materials used (stimuli, instructions, manipulations, and measures). Nowadays, such information can be shared via online repositories or platforms such as the Open Science Framework. Failing to do this impedes replications, and so we consider this another researcher DF during the reporting of studies, namely *R2: Failing to enable replication (re-running of the study).* Although both reproducibility and enabling replication are considered matters of reporting here, a preregistration of the study could already specifically and precisely indicate what information is going to shared and in what manner.

Furthermore, for preregistered studies, there exists an additional researcher DF related to reporting of results. Specifically, the researcher(s) could *R3: Fail to mention, misrepresent, or misidentify the study preregistration.* Studies of preregistrations of randomized clinical trials highlight that preregistrations in the medical literature are often not followed in the final report (Chan et al., 2004). This problem can be avoided by having reviewers compare the preregistration to the (submitted) research article.

Moreover, researchers could fail to present relevant unpublished work in their final publication. This creates *R4: Failing to report so-called “failed studies” that were originally deemed relevant to the research question*. Note that failed studies are often those that showed no statistically significant results, which is a main reason for authors for not publishing the results (Cooper et al., 1997). However, if the study was seen in advance as valid and methodologically rigorous, the study cannot be considered “failed” and should be considered as adding relevant evidence. This is the idea underlying the article format of registered reports, in which the rationale and methods of a study are reviewed and the final study is accepted for publication regardless of the (statistical significance of the) final result (de Groot, 1956/2014; Chambers, 2013; Simons et al., 2014).

There are two more researcher DFs in the reporting of studies that bear on the results or the rationale for the study, respectively. First, researcher(s) could *R5: Misreport results and p-values* (Bakker and Wicherts, 2011), for instance by presenting a statistically non-significant result as being significant. This practice and similar practices of misreporting of results (e.g., incorrectly stating a lack of moderation by demographic variables) are quite common (John et al., 2012; Nuijten et al., 2015). Second, researchers can choose to *R6: Hypothesize after the results are known (HARKing).* They can falsely present results of data explorations as though they were confirmatory tests of hypotheses that were stipulated in advance (Wagenmakers et al., 2011), which is related to lack of clear hypotheses (T1) and appears to be quite commonly practiced by psychologists (John et al., 2012). Both types of misreporting lower trust in reported findings and potentially also the replicability of results in later research.

## Discussion

We created a list of 34 researcher DFs, but our list is in no way exhaustive for the many choices that need be made during the different phases of a psychological experiment. Some of the researcher DFs are clearly related to others, but we nonetheless considered it valuable to list them separately according to the phase of the study. One can envision many other ways to create bias in studies, including poorly designed experiments with confounding factors, biased samples, invalid measurements, erroneous analyses, inappropriate scales, data dependencies that inflate significance levels, etc. Moreover, some of the researcher DFs on our list do not apply to other statistical frameworks and our list does not include the specific DF associated with those frameworks (e.g., specifying priors in Bayesian analyses). Here we focused on the researcher DFs that are often relevant even for well-designed and rigorously conducted experiments and other types of psychological studies that use NHST to test their hypotheses of interest.

We sympathize with Gelman and Loken’s (2014) argument that the term questionable research practices in relation to researcher’s use of researcher DFs is not always necessary, because the majority of the researcher DFs we describe involve choices that are arbitrary; researchers just need to decide between these different options and *could but not necessarily will* use these researcher DFs in an opportunistic manner. What matters is that the data could be collected and analyzed in different ways and that the final analyses reported in the research article could have been chosen differently if the results (based on these different choices and bearing on statistical significance) had come out differently. The issue, then, is not that all researchers try to obtain desirable results by exploiting researcher DFs but rather that the researcher DFs have strong potential to create bias. Such potential for bias is particularly severe for experiments that study subtle effects with relatively small samples. Hence, we need an appropriate way to deal with researcher DFs. One way to assess the relevance of choices is to report all potentially relevant analyses either as traditional sensitivity analyses or as a multiverse analysis (Steegen et al., 2016). Another solution is that the data are available for independent reanalysis after publication, although this is not always possible due to low sharing rates (Wicherts et al., 2011). However, preventing bias is better than treating it after it has occurred. Thus, the preferred way to counter bias due to researcher DFs is to preregister the study in a way that no longer allows researchers to exploit them.

The ideal preregistration of a study provides a *specific*, *precise*, and *exhaustive* story of the planned research, that is, it describes all steps, with only one interpretation, and excludes other possible steps. Our list can be used in research methods education, as a checklist to assess the quality of preregistrations, and to determine the potential for bias due to (arbitrary) choices in unregistered studies. Presently, we are conducting a study focusing on the quality of a random sample of actual preregistrations on the Open Science Framework in which we use a scoring protocol based on our checklist to assess the degree to which these preregistrations avoid any potential *p*-hacking. The protocol assesses the preregistration’s specificity, precision, and completeness at the level of each researcher DF; a score of 0 is assigned if the DF is not limited, whereas 1 and 2 are assigned if the description is partly or fully specific and precise, respectively. A score 3 is assigned if it is also exhaustive, i.e., if it excludes other steps. By applying the protocol, authors can also score their own preregistration, enabling them to improve their preregistration, and reviewers of registered reports and registered studies can use the protocol as well. Both authors and reviewers can thus use the protocol to limit potential *p*-hacking in planned studies.

We suggest a few avenues for future research. First, while most of the researcher DFs in our list are relevant to other statistical frameworks as well, the list should be adapted for studies planning to use confidence intervals and certain precision of effect size estimates (Cumming, 2012, 2014; Maxwell et al., 2015), or Bayesian analyses (Kruschke, 2015). Second, where we focused on preregistrations and assessing their quality, it is likewise urgent to develop and assess protocols for using ‘open materials,’ ‘open data,’ and ‘open workflows’ (Nosek et al., 2012). These transparent practices have many benefits and are currently gaining traction (e.g., Eich, 2014; Kidwell et al., 2016), but are often insufficiently detailed, documented or structured to allow other researchers to reproduce and replicate results (e.g., reuse of open data requires solid documentation and meta-data; Wicherts, 2017). While we believe all these open practices strengthen research, a lot can still be gained by creating protocols that provide specific, precise, and exhaustive descriptions of materials, data, and workflow.

## Author Contributions

JW, MB, CV, MvA conceived of the study idea. JW, MB, CV, MvA, HA, RvA wrote sections of the paper and made revisions. JW, MB, CV, MvA, HA, RvA approved final version of the paper.

## Conflict of Interest Statement

The authors declare that the research was conducted in the absence of any commercial or financial relationships that could be construed as a potential conflict of interest.
